# Effects of zinc-bearing palygorskite on rumen fermentation *in vitro*

**DOI:** 10.5713/ajas.17.0920

**Published:** 2018-04-19

**Authors:** Mengjiao Chen, Yumeng Xi, Lin Zhang, Hanfang Zeng, Yeqing Li, Zhaoyu Han

**Affiliations:** 1 Institute of Dairy Science, College of Animal Science and Technology, Nanjing Agricultural University, Nanjing 210095,China; 2 Animal Husbandry Institute, Jiangsu Academy of Agricultural Sciences, Nanjing 210014, China

**Keywords:** Zinc-bearing Palygorskite, Rumen Fermentation, *In vitro*, Dairy Cows

## Abstract

**Objective:**

The aim of the study was to investigate the effect of zinc-bearing palygorskite (Zn-Pal) on rumen fermentation by *in vitro* gas-production system.

**Methods:**

In trial, 90 incubators were evenly divided into five groups: control (0% Zn-Pal), treatment I (0.2% Zn-Pal), treatment II (0.4% Zn-Pal), treatment III (0.6% Zn-Pal), and treatment IV (0.8% Zn-Pal). The contents of zinc for treatments were 0, 49, 98, 147, 196 mg/kg, respectively. The main chemical composition and microstructure of Zn-Pal was investigated by X-ray diffraction. The physicochemical features were evaluated by Zeta potential analysis, cation-exchange capacity, ethylene blue absorption and specific surface area (the Brunauer–Emmett–Teller method). *In vitro* gas production (GP) was recorded at 3, 6, 9, 12, 18, 24, 36, 48, 60, and 72 h incubation. Incubation was stopped at 0, 6, 12, 24, 48, and 72 h and the inoculants were tested for pH, microbial protein yield (MCP), NH_3_-N, volatile fatty acids (VFAs), lipopolysaccharide (LPS).

**Results:**

The results showed that the GP in the treatment groups was not significantly different from the control groups (p>0.05). Compared to the control group, pH was higher at 24 h, 48 h (p<0.05), and 72 h (p<0.01) (range 6 to 7). The concentration of NH_3_-N in the three treatment groups was higher than in the control group at 24 h (p<0.01), meanwhile, it was lower at 48 h and 72 h (p<0.01), except in the treatment IV. The concentration of MCP in treatment I group was higher than in the control at 48 h (p<0.01). Compared with control, the LPS concentration in treatment III became lower at 12 h (p<0.05). Total VFAs in treatments were higher than in the control at 24 h, 48 h (p<0.05).

**Conclusion:**

These results suggest that the addition of Zn-Pal can improve the rumen fermentation, especially when adding 0.2% Zn-Pal.

## INTRODUCTION

Palygorskite (Pal) is a common clay mineral, which has a large surface area, a moderate layer charge, ion-exchange and adsorption ability, and rheological and catalytic properties with its special micropores and channels, fine particle size and fibrous habit [1] and these characters make it be applicable for use in many industries as well as animal nutrition [2]. Palygorskite can act as an adsorbent for toxins, bacteria and even viruses in the intestine [3], and as a protective coating for the stomach and intestines. Zaid et al [4] found that palygorskite was effective in the treatment of mild-to-moderate acute diarrhea in humans as an additional safe agent. Recently, a study reported that palygorskite was beneficial to intestinal integrity, which improved growth performance in weaned piglets [5].

In clay mineral, as the carrier, load some metal ions, such as Ag^+^, Cu^2+^, Zn^2+^ preparation of inorganic antibacterial agent has good antibacterial properties. Zinc has many physiological functions, not only is an essential trace mineral which is required for growth, enzyme structure, and function for poultry [6], but also can regulate microbial activity [7]. Zinc-bearing clinoptilolite could act as an antibacterial agent against *Salmonella*, inhibiting *Salmonella* growth and improving the gut health [8]. Additionally, Zn bearing zeolite and montmorillonite have been synthesized based on their high specific area and adsorption capacity [9]. It has been demonstrated that supplementation of these Zn bearing nonmetallic minerals can promote growth performance, improve intestinal morphology, inhibit the growth of pathogenic bacteria and enhance intestinal antioxidant capacity [9,10]. Moreover, they could be replaced antibiotics widely used in poultry production due to their antimicrobial ability.

However, aside from its being a potential antibiotic in poultry, little was known about the application of zinc-bearing palygorskite (Zn-Pal) in dairy cattle. In this study, Pal was used to prepare Zn-Pal through the ion-exchange method and evaluated the bioavailability of Zn-Pal as a substitute of antibiotic for dairy cattle with technology of *in-vitro*-rumen fermentation.

## MATERIALS AND METHODS

### Zn-Pal, experimental design, and *in vitro* fermentation

#### Preparation of Zn-Pal

Palygorskite was kindly provided by Jiangsu Sinitic Biotech Co., Ltd. (Xuyi, Jiangsu, China). The chemical composition of Pal clay mineral is SiO_2_ 53.73%, Al_2_O_3_ 10.90%, MgO 6.81%, CaO 3.08%, K_2_O 2.00%, and Fe_2_O_3_ 7.84% (Table 1). Zn-Pal was prepared according to the method described by Yan et al [11]. In detail, palygorskite was first calcinated at around 300°C for 3 h in a muffle oven. After cooling down, palygorskite was mixed with ZnCl_2_ (ZnCl_2_≥98.0%; 4:1, wt/wt) purchased from Nanjing Chemical Reagent Co., Ltd. (Nanjing, Jiangsu, China) in a stainless steel blade grinder. The mixture was subsequently calcinated at 300°C for 3 h in a muffle oven. After cooling to room temperature, the mixture was washed repeatedly by deionized water until there was no white deposition generated in the washed solution when swigged with 0.1 mol/L AgNO_3_ solution. Finally, the washed mixture was collected and dried at around 105°C for 2 h in an air oven, and then ground through a 200-mesh sieve after cooling down. The amount of Zn adsorbed by palygorskite was 24.5 mg/g with inductively coupled plasma mass spectrometry (ICP-MS, Optima 2100 DV, Perkin Elmer, Waltham, MA, USA) and according to the method described by Yan et al [11].

#### Characterization of Zn-Pal

The main chemical compositions of Pal and Zn-Pal were determined by a Minipal 4X-ray fluorescence spectrometer (PAN, Almelo, Netherlands). X-ray diffraction (XRD) patterns were collected on an X’pert PRO X-ray power diffractometer, which equipped with a Cu-Kα radiation source (40 kV, 40 mA) from 3° to 80° (2θ) at a scanning step time of 15.2 s, with a step interval about 0.017°, divergence slit of 0.5°. The physico-chemical properties of Pal and Zn-Pal were shown in Table 2. Zeta potential of Pal and Zn-Pal were measured by a Malvern Zetasizer Nano system (Malvern Instruments, Malvern, UK) at 25°C, using a folded capillary cell. Before measurements, a sample of 0.50 g of obtained palygorskite was fully dispersed in 100 mL of distilled water under high-speed stirring at 11,000 rpm for 20 min. The specific surface area is determined by the Brunauer–Emmett–Teller (BET) method. Determinations of cation-exchange capacity and ethylene blue absorption were performed according to the method described by Qiao et al [12].

#### Experimental design

The substrate used was a common total mixed ration diet (with a 50:50 forage: concentrate diet, 54.6% dry matter, 17.15% crude protein, and 34.72% neutral detergent fiber, which was dried at 65°C for 48 h and broken up by passing it through a 1-mm screen. The composition and nutrient content of the basal diet are presented in Table 3.

Experiments were conducted to measure *in vitro* gas production (GP) characteristics of Zn-Pal. Inclusive levels of Zn-Pal were 0% (Control, n = 18), 0.2% (Treatment I, n = 18), 0.4% (Treatment II, n = 18), 0.6% (Treatment III, n = 18), 0.8% (Treatment IV, n = 18) *in vitro* incubation fluid, respectively. The Zn-Pal was mixed with the substrate before the commencement of the experiment. The pressure in the bottle was recorded at 3, 6, 9, 12, 18, 24, 36, 48, 60, and 72 h during the processes of *in vitro* fermentation. At 0, 6, 12, 24, 48, and 72 h, fluid was sampled to determine pH, NH_3_-N, volatile fatty acid (VFA), microbial protein yield (MCP), and lipopolysaccharide (LPS).

#### Substrate, inoculum and incubation

All animals involved in this study were cared for according to the principles of Nanjing Agricultural University Animal Care and Use Committee. Rumen fluid was collected from 3 healthy dairy cows (650 kg of mean body weight), which were killed after a 7-day adaptation to the diet. Ruminal fluid was collected from different locations of rumen, and then mixed and strained through four layers of cheesecloth into a pre-warmed thermos flask. The 100 mL of rumen fluid-buffer mixture and rumen fluid in the ratio of 4 to 1, was dispensed anaerobically into bottles containing 1 g of total mixed ration and additives 0%, 0.2%, 0.4%, 0.6%, 0.8% Zn-Pal. The composition and dosage of the rumen fluid-buffer mixture are performed according to the method described by Theodorou et al [13]. The serum bottles were filled with O_2_-free CO_2_ gas, and then capped with a rubber stopper. The bottles were kept in an incubator (JSGI-250T, JSR, Gongju, Korea) at 39°C.

Measurement of *in vitro* fermentation parameters: During the incubation, the GP kinetics were recorded at 3, 6, 9, 12, 18, 24, 36, 48, 60, and 72 h. Cumulative GP data were fitted to the model of McDonald [14] as follows:

$$\text{Y}=\text{A }(1-{\text{e}}^{-\text{c }(\text{t}-\text{L})})$$

Where Y is the volume of GP (mL per 100 mg DM) at time t, A is GP from soluble and insoluble fraction, c is the rate of GP, L the lag time (h) and t is the incubation time (h).

After incubation at 39°C for 0, 6, 12, 24, 48, 72 h, fluid was sampled to determine pH, NH_3_-N, VFA, MCP, and LPS. The pH value of *in vitro* fermentation liquid was determined using a pH meter (Model PHS-3C, Shanghai Precision & Scientific Instrument Co., LTD, Shanghai, China). NH_3_-N content was determined by using a spectrophotometer (8500II, Thermo Electron Corporation, Waltham, MA, USA). MCP content was determined based on Lowry’s assay, with modifications described in Makkar [15], and bovine serum albumin was used as a standard. The levels of LPS were measured as described by Emmanuel et al [16] using the Limulus amoebocyte lysate assay (Xiamen Houshiji, Ltd., Xiamen, China). To determine VFAs, 1 mL of fermentation medium was placed in a centrifuge tube, mixed uniformly with 0.2 mL of 25% metaphosphoric acid using crotonic acid as an internal standard, and then centrifuged at 12,000×g for 10 min. The supernatant was decanted into another test tube, capped, and stored in a refrigerator at 4°C until being analyzed with gas chromatography (GC-14B; Shimadzu, Kyoto, Japan). Subsamples were injected into a 30 m×0.32 mm×0.25 μm capillary. The temperatures of the detector, column and vaporization were 220°C, 130°C, and 180°C, respectively. Nitrogen was used as a carrier.

### Statistical analysis

Data were analyzed using general linear models followed by Duncan’s multiple range tests, on Statistical Analysis System Institute. Treatment, fermentation time, and the treatment× fermentation time interaction were the fixed effects. Values are presented as least squares mean±standard error of mean, and the differences were considered significant when p<0.05 and very significant when p<0.01.

## RESULTS

### Characterization of Zn-Pal

**Figure 1:**
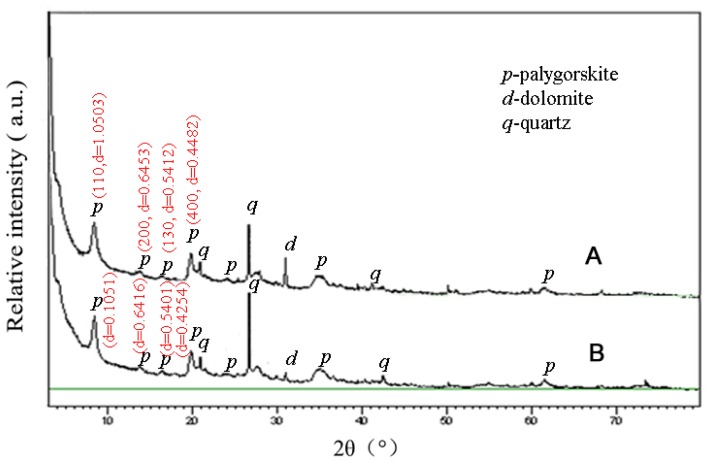
X-ray diffraction patterns of natural palygorskite (A) and zinc-bearing palygorskite (B). The raw palygorskite with the characteristic reflections appeared at 2θ = 8.41° (110, d = 1.0503 nm), 2θ = 13.72° (200, d = 0.6453 nm), 2θ = 16.37° (130, d = 0.5412 nm), and 2θ = 19.81° (400, d = 0.4482 nm), respectively. The heated palygorskite with the characteristic reflections appeared at 2θ = 8.47° (110, d = 1.0434 nm), 2θ = 13.71° (200, d = 0.6457 nm), 2θ = 16.42° (130, d = 0.5398 nm), and 2θ = 19.86° (400, d = 0.4470 nm), respectively. *p* = palygorskite, *d* = dolomite, *q* = quartz.

The X-ray diffraction patterns of the Pal and Zn-Pal samples are shown in Figure 1. The palygorskite with the characteristic reflections appeared at 2θ = 8.41° (d = 1.0503 nm, 110 crystal plane), 2θ = 13.72° (d = 0.6453 nm, 200 crystal plane), 2θ = 16.37° (d = 0.5412 nm, 130 crystal plane), and 2θ = 19.81° (d = 0.4482 nm, 400 crystal plane), respectively. And the Zn-Pal with the characteristic reflections appeared at 2θ = 8.41° (d = 1.0510 nm, 110 crystal plane), 2θ = 13.80° (d = 0.6415 nm, 200 crystal plane), 2θ = 16.41° (d = 0.5400 nm, 130 crystal plane), and 2θ = 19.80° (d = 0.4819 nm, 400 crystal plane), respectively.

Table 1 shows the chemical composition of Pal and Zn-Pal. After being modified with ZnCl2, the content of Zn2+ ions of Zn-Pal increased, which is similar with the result by method of inductively coupled plasma mass spectrometry. It can be seen from Table 2 that the BET surface area of Pal is 164.29 m^2^/g, the cation exchange capacity of Pal is 28.01 mmol/100 g, the Zeta potential of Pal is −13.6 mV, and the BET surface area of Zn-Pal is 136.60 m^2^/g, the cation exchange capacity of Zn-Pal is 35.52 mmol/100 g, the Zeta potential of Zn-Pal is −15.5 mV.

### Effect on gas production kinetics

Effect of Zn-Pal on GP at different times *in vitro* incubation is given in Table 4. While the other treatments became lower than that of the control staring at 3 h, the GP of the treatment I became higher at 9 h (p>0.05) and the gap tended to be stable with the extension of fermentation time, though no difference was shown between control and treatment groups. In addition, compared with treatment I, the GP of the treatment II, treatment III, and treatment IV became lower at 18, 24, 36, 48, 60, and 72 h (p<0.05) except the GP of the treatment IV has no significant difference at 60 and 72 h (p>0.05), meanwhile the GP of the treatment II has most significant difference at 24 and 36 h (p<0.01).

### *In vitro* rumen fermentation parameters

Effects of Zn-Pal on pH, NH_3_-N, MCP, and LPS profile values at different times of *in vitro* incubation are given in Table 5. Effects of Zn-Pal on VFAs profile value at different times of *in vitro* incubation are given in Table 6. Under this experiment, from 0 to 24 h, the pH was linearly decreased with the extension time of incubation and from 24 to 72 h, the pH was linearly increased with the extension time of incubation. The pH in the treatment IV became lower than in the control group at 6 h (p<0.05), while pH became higher at 24, 48, and 72 h (p<0.05, range 6 to 7), though no difference was shown between control and other treatment groups at 0 and 12 h (p>0.05).

The concentration of NH_3_-N from 0 to 12 h was found no differences among the control and treatment groups (p>0.05). The concentration of NH_3_-N in the treatment I, treatment II, and treatment III groups were higher than in the control group at 24 h (p<0.01), and the three treatment groups were lower than in the control group at 48 and 72 h (p<0.01), though no different significant was shown between control and other treatment groups at 24, 48, and 72 h (p>0.05).

The MCP synthesis in the treatment I was lower than control (p<0.05) and the MCP synthesis in the treatment III was higher than control (p<0.05), meanwhile, though no difference was shown between control and other treatments at 6 h. The MCP synthesis was significantly increased in the treatment I than that of control at 48 h (p<0.01), while other treatments also increased compared to the control at 12, 48, and 72 h, but there had no significant difference between them (p>0.05).

The concentration of LPS in the treatment I and treatment II were increased than control at 6 h (p<0.05). However, treatment III produced the lower concentration of LPS at 12 h compared to the control (p<0.05). The LPS concentration in treatment I was lower than control at 24, 48, and 72 h (p> 0.05), while other treatments was higher than control, although there had varying degrees of increase.

Acetate and total VFA in all treatments were higher than the control at all times, while there had significant difference at 24, 48, and 72 h (p<0.05). The amount of butyrate in treatment III were significantly lower than in the control at 6 h (p<0.05), while the level of propionate in all treatments were significantly higher than in the control at 24 h (p<0.05).

## DISCUSSION

As shown in the XRD patterns (Figure 1), there is no obvious difference among the scattering peaks from the two samples, and only the intensity of reflections at 1.05, 0.54, and 0.45 nm weaken slightly, because of the removal of partial zeolite water from the tunnel of palygorskite during thermal treatment process [17], indicating that zinc-bearing can keep the basic crystal structure of palygorskite. Moreover, there is no new crystal phase generates during modification process, which reveals that Zn^2+^ ions interact with palygorskite by an ion-exchange process. The change of BET surface area and cation exchange capacity is due to the process of zinc-bearing. The change of zeta potential is due to the dissociation and dispersion of palygorskite crystal beam and the exchange of sodium ions with metal ions on the surface of palygorskite.

In current study, the Zeta potentials of Pal and Zn-Pal samples are all negative and became more negative after Zn-bearing. The increase of negative charges is favorable to enhance the affinity of palygorskite with cationic matters, e.g., cationic dyes, and then improve the adsorption for them. Marchuk et al [18] have proved that palygorskite particle could be considered as an anion with large size and high charge density, which attracts the ions of opposite sign and repels the ions of the same sign of the particle). Hamieh et al [19] have proved that the negatively charged rod-crystals were surrounded by opposite ions and the surface potential of particle decreased, when palygorskite was dispersed in various solutions containing inorganic ions. The thickness of the diffuse layer might reduce due to increase in the concentration of electrolyte, while at the same ion valency [20]. Therefore, the difference in the physico-chemical property and the amount of Zn^2+^ of Zn-Pal might indicate different effects in rumen fermentation.

The GP is a comprehensive indicator for reflecting the degree of ruminal fermentation. GP has been proven to be positively related to the activity of ruminal bacteria [21]. Meanwhile, higher microbial activity and GP are often accompanied by a stronger fermentation level of fodder. As the fermentation time goes on, the GP in the treatment groups was increased first and then decreased, possibly because the nutrients in the substrate were beneficial to the growth of bacteria, the number of fungi gradually decreased until disappeared, resulting in an increase in GP; fermentation substrates are limited, limiting the number and activity of bacteria and thus resulting in reduced GP. In current study, the GP of the treatment I became higher starting at 9 h. The result indicated that a low dose of Zn-Pal increased the GP in the rumen, which is beneficial for the health of the ruminant. A high level of Zn-Pal has no significant effect on GP. The main reason might be that Zeta potential of Zn-Pal becomes more negative, which promotes the adsorption of Zn-Pal.

pH is an important parameter that keeps the balance of rumen microbes and reflects the extent and pattern of diet fermentation. Normal ruminal pH values range from 6 to 7. If the value is too low or too high, is not conducive to the growth of rumen microorganisms, which greatly affect the physiological function of animals. In general, rumen pH decreased after feeding, and then gradually increased, ruminal pH fluctuated and changes, pH fluctuations in the curve reflects the concentration of organic acids and produce saliva changes. In current study, the pH in the treatment groups became higher than in the control group at 24, 48, and 72 h. Meanwhile, all values were in the normal range. This result indicated that adding Zn-Pal was beneficial to dairy cows by increasing the ruminal pH. This finding is consistent with the result of Ehrlich et al [22], who reported that adding 4% sodium bentonite or 2% zeolite could also increase pH and improve the ruminal health of cows. In addition, the ion-exchange property of the clay could alter the pH and the ionic composition (including trace elements) of the gastrointestinal fluids, resulting in enhanced enzymatic activity, and this is beneficial for ruminal microbial fermentation [23]. Then, the Zn-Pal increased the pH in the rumen, which is beneficial for the health of the ruminant.

Ammonia concentration in the rumen is a balance between degradation of feed protein and uptake of ammonia for synthesis of microbial protein. In the rumen microflora, about 80% of the bacteria treat ammonia nitrogen as a direct nitrogen source. In this study, adding Zn-Pal could promote the release of NH_3_-N and the synthesis of MCP. Kennedy [7] have reported that adding the content of Zn in diet can reduce the concentration of NH_3_-N, promote cell protein synthesis. In addition, Kardaya [24] have suggested that nanoporous structure of zeolite could cause the release of ammonia and maintain the stability of ammonia concentrations because of their sustained release and cation-exchange capacity. In the present study, Zn-Pal has more cation-exchange capacity than palygorskite. This finding is consistent with the result of Zeng et al [25], who reported that adding 3% palygorskite or heated palygorskite *in vitro* was beneficial for nitrogen use and MCP synthesis. It has also proved that milk protein yield increased with 10 kg/t of supplemental dietary palygorskite in diets [26]. Therefore, adding Zn-Pal was beneficial for nitrogen use and MCP synthesis, and the Zn-Pal showed better stability than the palygorskite. In addition, Zn-Pal is rich in Zn^2+^, Al^3+^, and Mg^2+^ and has a strong cation-exchange capacity, which might affect the corresponding ion concentrations in rumen. It has also been proven that supplemental 23 g/kg of zeolite in the cow diet can improve ruminal Al^3+^ concentrations [27].

The VFAs are the products of microbial fermentation of carbohydrates in the rumen and represent the main supply of metabolizable energy for ruminants [28]. In this study, acetate and total VFA were increased by supplementing Zn-Pal. Palygorskite has similarity structure and biological characteristics with natural silicate, as do minerals such as bentonite, zeolite and montmorillonite. Salem et al [29] reported the addition of 12 g/d of bentonite to the sheep diet resulted in an increase of total VFA. Zeng et al [25] showed palygorskite and heated palygorskite supplementation increased VFA concentrations in the rumen. All of these results indicate that silicate could improve rumen fermentation and VFA formation. In the present study, the Zeta potential of Zn-Pal becomes more negative, which promotes the adsorption of palygorskite. Therefore, Zn-Pal has a higher adsorption capacity than palygorskite, which could provide a stable internal environment in which more beneficial bacteria exist, improving carbohydrate decomposition and increasing VFAs [30].

The LSP is the product of the cell wall of Gram-negative bacteria, which are the predominant bacterial group in the rumen. Plaizier et al [31] has revealed that a decline in ruminal pH causes the death and cell lysis of gram-negative bacteria, resulting in an increase in the free ruminal LPS concentration. In addition, the increased in the free ruminal LPS concentration may also be caused by the rapid growth of bacteria. In current study, compared with control, the LPS concentration in treatment I rised first and then lower with time, meanwhile, the LPS concentration in other treatment groups become lower at 12 and 24 h. Zinc is the body of the necessary trace elements, but also has a certain antibacterial propertie [32]. The release of Zn^2+^ from the surface or the pores of the earth is negatively charged with a negatively charged microbial cell membrane that destroys the activity of the cell synthase and interferes with the DNA synthesis, resulting in the loss of the microorganism and the ability to divide and proliferate [33] Abend et al [34] have proved that montmorillonite has a unique charge characteristic, which has a lasting effect. Researcher has found that adding 0.2% montmorillonite to the diet can reduce the LPS concentration in digestive segments of chyme, except for cecum [35].

## CONCLUSION

In conclusion, the zinc-bearing treatment process not merely maintained the crystal structure of palygorskite, but also increased the cation exchange and the content of zinc ion. Addition of Zn-Pal can improve rumen fermentation, promote VFA formation, MCP synthetic, and LPS adsorb by promoting the release of Zn, especially when adding 0.2% Zn-Pal.

## Figures and Tables

**Table 1:** The chemical composition of the palygorskites

Item	Palygorskite	Zinc-bearing palygorskite
SiO_2_ (%)	53.73	53.07
Al_2_O_3_ (%)	10.90	10.43
Fe_2_O_3_ (%)	7.84	7.75
MgO (%)	6.81	6.11
CaO (%)	3.08	1.85
K_2_O (%)	2.00	1.92
Zn (ppm)	112.25	27,198.47

The chemical composition of natural and zinc-bearing palygorskite were determined by a MiniPal 4 X-ray fluorescence spectrometer (PANanalytical Co., Almelo, the Netherlands).

**Table 2:** Physico-chemical properties of the palygorskites

Items	Palygorskite	Zinc-bearing palygorskite
BET surface area (m^2^/g)	164.29	136.60
Cation exchange capacity (mmol/100 g)	28.01	35.52
Ethylene blue absorption (mmol/100 g)	15	15
Zeta potential (mV)	−13.6	−15.5

BET, Brunauer–Emmett–Teller.

These data were measured by Qiao et al [12]. Zeta potential was measured by a Malvern Zetasizer Nano system (Malvern Instruments, Malvern, UK).

**Table 3:** Composition and nutrient levels of basal diet in dairy cows after parturition (DM basis) %

Ingredients	Content
Corn	20.86
Barley	1.89
Soybean meal	12.49
Cottonseed	3.41
Primix1)	2.70
DDGS	6.24
Wheat silage	20.75
Alfalfa hay	12.13
Chinensis wildrye	4.04
Beet meal	12.26
Brewers grain, wet	3.23
Nutrient levels	
ME (MJ/kg)	10.71
NEL (MJ/kg)	6.89
CP	18.32
EE	4.33
Ash	9.22
ADF	22.81
NDF	34.58
Ca	0.41
P	0.38

DM, dry material; DDGS, distillers dried grains with solubles; ME, metabolic energy; NE_L_, location of net energy; CP, crude protein; EE, ethyl ether extract; ADF, acid detergent fiber; NDF, neutral detergent fiber.

1) Content of per kilogram premix: 500 kIU vitamin A, 140 kIU vitamin D_3_, 2,000 kIU vitamin E, 2,200 mg Cu, 4,000 mg Fe, 2,400 mg Mn, 5,600 mg Zn, 80 mg I, 35 mg Se, and 20 mg Co.

**Table 4:** Influence of zinc-bearing palygorskite on *in vitro* gas production

Time (h)	Control	Treatment I	Treatment II	Treatment III	Treatment IV	SEM	p-value1)		

							T	F	T×F
3	39.70	36.67	37.37	37.80	38.30	0.50	<0.001	<0.001	<0.001
6	80.53	79.87	75.80	77.43	77.75	1.01			
9	129.70	132.63	124.67	125.77	125.75	1.40			
12	172.40	177.13	167.90	169.10	170.10	1.41			
18	213.27^ab^	220.20^a^	206.87^b^	209.50^b^	210.15^b^	1.67			
24	243.80^ab^	251.83^a^	236.00^b^	239.43^b^	240.00^b^	1.90			
36	269.30^ab^	279.33^a^	261.17^b^	264.57^b^	266.50^b^	2.20			
48	286.07^ab^	296.73^a^	278.57^b^	281.47^b^	283.50^b^	2.26			
60	296.87^ab^	306.80^a^	289.00^b^	291.47^b^	294.90^ab^	2.28			
72	301.87^ab^	312.73^a^	294.00^b^	296.87^b^	300.10^ab^	2.37			

SEM, standard error of the mean.

1) T, effect of treatments; F, effect of fermentation time; T×F, interaction effect of treatments and fermentation time.

a,b Within the same row with different lower-case letters mean significant at p<0.05.

**Table 5:** Influence of zinc-bearing palygorskite on rumen fermentation parameters

Item	Time	Control	Treatment I	Treatment II	Treatment III	Treatment IV	SEM	p-value1)		

								T	F	T×F
pH	6	6.68^a^	6.66^a^	6.65^ab^	6.60^ab^	6.56^b^	0.02	0.381	<0.001	0.018
	12	6.42	6.39	6.41	6.43	6.41	0.01			
	24	6.26^a^	6.32^b^	6.31^b^	6.33^b^	6.32^b^	0.01			
	48	6.38^a^	6.45^b^	6.50^b^	6.45^b^	6.44^b^	0.02			
	72	6.49^A^	6.62^B^	6.62^B^	6.62^B^	6.65^B^	0.02			
NH_3_-N (mg/dL)	6	8.22	8.05	8.65	8.25	8.63	0.12	0.144	<0.001	<0.001
	12	8.27	8.09	8.28	7.82	7.86	0.08			
	24	9.62^b^	11.54^a^	11.56^a^	11.60^a^	11.26^a^	0.24			
	48	5.62^a^	4.09^c^	4.64^b^	4.72^b^	5.12^ab^	0.16			
	72	6.70^a^	4.01^c^	4.99^b^	5.13^b^	6.36^a^	0.29			
MCP (mg/dL)	6	220.82^ab^	221.20^b^	222.77^ab^	232.14^a^	220.82^ab^	2.56	0.047	<0.001	0.037
	12	217.53	228.61	223.62	228.86	222.40	0.88			
	24	235.43	240.18	237.99	230.81	238.36	1.44			
	48	245.79^b^	261.25^a^	247.12^b^	254.80^ab^	257.48^a^	1.94			
	72	247.37	264.66	262.59	265.52	265.64	3.12			
LPS (EU/L)	6	353.96^b^	479.65^a^	459.75^a^	393.77^ab^	444.98^ab^	17.04	0.283	0.051	0.039
	12	468.24^a^	453.71^a^	418.37^ab^	338.53^b^	375.65^ab^	18.71			
	24	432.01	407.57	425.88	426.49	412.91	12.25			
	48	415.91^bc^	382.81^c^	497.99^ab^	538.47^a^	484.01abc	19.78			
	72	395.59^B^	359.88^B^	517.14^A^	555.22^A^	513.58^A^	22.00			

SEM, standard error of the mean; MCP, microbial protein yield; LPS, lipopolysaccharide.

1) T, effect of treatments; F, effect of fermentation time; T×F, interaction effect of treatments and fermentation time.

a–c Within the same row with different lowercase letters mean significant at p<0.05.

A,B Within the same row with different capital letters mean significant at p<0.01.

**Table 6:** Influence of zinc-bearing palygorskite on the production of VFA (mM/L)

Item	Time	Control	Treatment I	Treatment II	Treatment III	Treatment IV	SEM	p-vale1)		

								T	F	T×F
Acetate	6	17.65^a^	18.37^ab^	18.66^ab^	19.91^b^	19.36^b^	0.21	<0.001	<0.001	<0.001
	12	19.65	20.37	21.66	21.91	21.36	0.23			
	24	22.27	22.06	23.46	23.59	22.53	0.32			
	48	23.90^b^	26.12^a^	26.54^a^	27.40^a^	26.79^a^	0.90			
	72	21.01^b^	25.41^a^	24.88^a^	23.42^ab^	23.77^ab^	0.32			
Propionate	6	5.99	5.96	5.90	6.27	5.96	0.19	<0.001	<0.001	<0.001
	12	6.69	7.26	6.90	6.87	7.26	0.11			
	24	6.02^b^	7.90^a^	7.44^ab^	8.18^a^	7.43^ab^	0.20			
	48	7.93	8.87	8.72	8.00	8.36	0.20			
	72	8.23	8.22	9.13	9.05	8.94	0.14			
Butyrate	6	1.15^a^	0.80^a^	0.72^a^	0.31^b^	0.70^a^	0.07	<0.001	<0.001	<0.001
	12	1.74	1.80	1.74	1.67	1.79	0.08			
	24	2.86	2.79	2.57	2.72	2.71	0.20			
	48	3.20	3.11	3.25	3.05	3.16	0.06			
	72	3.24	3.13	3.14	3.25	3.17	0.02			
TVFA	6	28.37^a^	31.88^ab^	31.06^ab^	33.02^b^	32.79^b^	0.45	<0.001	<0.001	<0.001
	12	32.29^b^	34.26^ab^	35.31^a^	35.66^a^	35.59^a^	0.34			
	24	33.77^b^	39.66^a^	40.27^a^	41.66^a^	39.62^a^	0.73			
	48	43.029^b^	46.96^a^	47.28^a^	46.97^a^	48.35^a^	1.28			
	72	41.38^b^	42.96^b^	46.35^a^	46.88^a^	44.42^b^	0.48			

VFA, volatile fatty acids; SEM, standard error of the mean; TVFA, total volatile fatty acids.

1) T, effect of treatments; F, effect of fermentation time; T×F, interaction effect of treatments and fermentation time.

a,b Within the same row with different lowercase letters mean significant at p<0.05.

## References

[1] Xu J, Wang W, Mu B, Wang A (2012). Effects of inorganic sulfates on the microstructure and properties of ion-exchange treated palygorskite clay. Colloids Surf A Physicochem Eng Asp.

[2] Chalvatzi S, Kalamaki MS, Arsenos G, Fortomaris P (2016). Dietary supplementation with the clay mineral palygorskite affects performance and beneficially modulates cecal microbiota in laying pullets. J Appl Microbiol.

[3] Reynolds JEF (1982). Martindale: the extra pharmacopoeia.

[4] Zaid MR, Hasan M, Khan AA (1995). Attapulgite in the treatment of acute diarrhoea: a double-blind placebo-controlled study. J Diarrhoeal Dis Res.

[5] Zhang J, Lv Y, Tang C, Wang X (2013). Effects of dietary supplementation with palygorskite on intestinal integrity in weaned piglets. Appl Clay Sci.

[6] Fischer Walker C, Black RE (2004). Zinc and the risk for infectious disease. Annu Rev Nutr.

[7] Kennedy DW, Bunting LD (1991). Alterations in ruminal utilization of magnesium and zinc in lambs fed different ratios of concentrate: forage. Int J Vitam Nutr Res.

[8] Wang LC, Zhang TT, Wen C (2012). Protective effects of zinc-bearing clinoptilolite on broilers challenged with *Salmonella pullorum*. Poult Sci.

[9] Hu CH, Qian ZC, Song J, Luan ZS, Zuo AY (2013). Effects of zinc oxide-montmorillonite hybrid on growth performance, intestinal structure, and function of broiler chicken. Poult Sci.

[10] Tang ZG, Wen C, Wang LC, Wang T, Zhou YM (2014). Effect of zinc-bearing zeolite clinoptilolite on growth performance, nutrient retention, digestive enzyme activities, and intestinal function of broiler chickens. Biol Trace Elem Res.

[11] Yan R, Zhang L, Yang X, Wen C, Zhou Y (2016). Bioavailability evaluation of zinc-bearing palygorskite as a zinc source for broiler chickens. Appl Clay Sci.

[12] Qiao L, Chen Y, Wen C, Zhou Y (2015). Effects of natural and heat modified palygorskite supplementation on the laying performance, egg quality, intestinal morphology, digestive enzyme activity and pancreatic enzyme mRNA expression of laying hens. Appl Clay Sci.

[13] Theodorou MK, Williams BA, Dhanoa MS, Mcallan AB, France J (1994). A simple gas production method using a pressure transducer to determine the fermentation kinetics of ruminant feeds. Anim Feed Sci Technol.

[14] Mcdonald I (1981). A revised model for the estimation of protein degradability in the rumen. J Agric Sci.

[15] Makkar HP, Sharma OP, Dawra RK (1982). Simple determination of microbial protein in rumen liquor. J Dairy Sci.

[16] Emmanuel DGV, Dunn SM, Ametaj BN (2008). Feeding high proportions of barley grain stimulates an inflammatory response in dairy cows. J Dairy Sci.

[17] Wang W, Li A, Zhang J, Wang A (2007). Study on superabsorbent composite. XI. Effect of thermal treatment and acid activation of attapulgite on water absorbency of poly (-acrylic acid)/attapulgite superabsorbent composite. Polym Compos.

[18] Marchuk A, Rengasamy P (2011). Clay behaviour in suspension is related to the ionicity of clay–cation bonds. Appl Clay Sci.

[19] Hamieh T, Siffert B (1994). Theoretical and experimental study of the surface charge density and the surface potential of coal—water suspensions in dissymmetrical electrolytes. Colloids Surf A Physicochem Eng Asp.

[20] Rusmin R, Sarkar B, Biswas B (2016). Structural, electrokinetic and surface properties of activated palygorskite for environmental application. Appl Clay Sci.

[21] Kang S, Wanapat M (2013). Using plant source as a buffering agent to manipulating rumen fermentation in an *in vitro* gas production system. Asian-Australas J Anim Sci.

[22] Ehrlich WK, Davison TM (1997). Adding bentonite to sorghum grain-based supplements has no effect on cow milk production. Aust J Exp Agric.

[23] Martínez ME, Ranilla MJ, Tejido ML, Ramos S, Carro MD (2010). Comparison of fermentation of diets of variable composition and microbial populations in the rumen of sheep and Rusitec fermenters. I. Digestibility, fermentation parameters, and microbial growth. J Dairy Sci.

[24] Kardaya D, Sudrajat D, Dihansih E (2012). Efficacy of dietary urea-impregnated zeolite in improving rumen fermentation characteristics of local lamb. Media Peternakan.

[25] Zeng HF, Lin LJ, Xi YM, Han ZY (2017). Effects of raw and heated palygorskite on rumen fermentation *in vitro*. Appl Clay Sci.

[26] Bampidis VA, Christodoulou V, Theophilou N, Kotsampasi V (2014). Effect of dietary palygorskite on performance and blood parameters of lactating Holstein cows. Appl Clay Sci.

[27] Grabherr H, Spolders M, Furll M (2009). Effect of several doses of zeolite A on feed intake, energy metabolism and on mineral metabolism in dairy cows around calving. J Anim Physiol Anim Nutr.

[28] Van SPJ, Fadel J, Sniffen CJ Discount factors for energy and protein in ruminant feeds [Formulation of diets].

[29] Salem FA, Soliman AS, Abdelmawla SM, Elmahdy MRM (2000). Effect of some feed additives to rations of growing sheep on growing performance, rumen fermentation, blood constituents and carcass characteristics. Annu Agric Sci Mosh.

[30] Bento CB, Azevedo AC, Gomes DI (2016). Effect of protein supplementation on ruminal parameters and microbial community fingerprint of Nellore steers fed tropical forages. Animal.

[31] Plaizier JC, Krause DO, Gozho GN, Mcbride BW (2008). Subacute ruminal acidosis in dairy cows: the physiological causes, incidence and consequences. Vet J.

[32] Bun SD, Guo YM, Guo FC, Ji FJ, Cao H (2011). Influence of organic zinc supplementation on the antioxidant status and immune responses of broilers challenged with eimeria tenella. Poult Sci.

[33] Sanchezsalcedo S, Shruti S, Salinas AJ (2014). *In vitro* antibacterial capacity and cytocompatibility of SiO_2_-CaO-P_2_O_5_ meso-macroporous glass scaffolds enriched with ZnO. J Mater Chem B.

[34] Abend S, Lagaly G (2000). Sol-gel transitions of sodium montmorllonite dispersions. Aappl Clay Sci.

[35] Zhou J, Dong G, Ao C (2014). Feeding a high-concentrate corn straw diet increased the release of endotoxin in the rumen and pro-inflammatory cytokines in the mammary gland of dairy cows. BMC Vet Res.

